# Outcomes and Pattern of Care for Spinal Myxopapillary Ependymoma in the Modern Era—A Population-Based Observational Study

**DOI:** 10.3390/cancers16112013

**Published:** 2024-05-25

**Authors:** Chenyang Wang, Michael K. Rooney, Christopher Alvarez-Breckenridge, Thomas H. Beckham, Caroline Chung, Brian S. De, Amol J. Ghia, David Grosshans, Nazanin K. Majd, Mary F. McAleer, Susan L. McGovern, Robert Y. North, Arnold C. Paulino, Subha Perni, Jay P. Reddy, Laurence D. Rhines, Todd A. Swanson, Claudio E. Tatsui, Martin C. Tom, Debra N. Yeboa, Jing Li

**Affiliations:** 1 Department of Radiation Oncology, The University of Texas MD Anderson Cancer Center, Houston, TX 77030, USA; mkrooney@mdanderson.org (M.K.R.); dgrossha@mdanderson.org (D.G.); apaulino@mdanderson.org (A.C.P.); mtom@mdanderson.org (M.C.T.); jing.li@mdanderson.org (J.L.); 2 Department of Neurosurgery, The University of Texas MD Anderson Cancer Center, Houston, TX 77030, USAlrhines@mdanderson.org (L.D.R.);; 3 Department of Neuro-Oncology, The University of Texas MD Anderson Cancer Center, Houston, TX 77030, USA

**Keywords:** SEER, myxopapillary ependymoma, surgical resection, radiotherapy

## Abstract

**Simple Summary:**

Myxopapillary ependymoma (MPE) is a rare tumor that typically grows in the lower spine. It is usually not aggressive and is most effectively treated with surgery. We studied data from over a thousand patients with spinal MPE from 2000 to 2020. Surgery was the main treatment for almost all patients. We found that younger age and having surgery were linked to better survival. Surprisingly, being male was associated with worse survival, and they tended to be diagnosed at a younger age than females. Our study, the largest of its kind, provides important information for treating spinal MPE.

**Abstract:**

(1) Background: Myxopapillary ependymoma (MPE) is a rare tumor of the spine, typically slow-growing and low-grade. Optimal management strategies remain unclear due to limited evidence given the low incidence of the disease. (2) Methods: We analyzed data from 1197 patients with spinal MPE from the Surveillance, Epidemiology, and End Results (SEER) database (2000–2020). Patient demographics, treatment modalities, and survival outcomes were examined using statistical analyses. (3) Results: Most patients were White (89.9%) with a median age at diagnosis of 42 years. Surgical resection was performed in 95% of cases. The estimated 10-year overall survival was 91.4%. Younger age (hazard ratio (HR) = 1.09, *p* < 0.001) and receipt of surgery (HR = 0.43, *p* = 0.007) were associated with improved survival. Surprisingly, male sex was associated with worse survival (HR = 1.86, *p* = 0.008) and a younger age at diagnosis compared to females. (4) Conclusions: This study, the largest of its kind, underscores the importance of surgical resection in managing spinal MPE. The unexpected association between male sex and worse survival warrants further investigation into potential sex-specific pathophysiological factors influencing prognosis. Despite limitations, our findings contribute valuable insights for guiding clinical management strategies for spinal MPE.

## 1. Introduction

Myxopapillary ependymoma (MPE) is a rare subtype of ependymoma characteristically arising from the ependymal cells of the filum terminale [1]. These tumors are histopathologically defined as grade II according to the most recent World Health Organization (WHO) classification and typically develop in the lumbosacral spine, although they may occur in other regions of the neuroaxis as well [2,3]. Spinal MPEs generally exhibit a slow, non-infiltrative growth pattern and thus carry a favorable prognosis. However, they may occasionally develop distant metastases or possess higher grade histological features, increasing the risk of compromised treatment outcomes and survival [4,5,6,7,8,9].

MPEs are extremely rare, with an estimated annual incidence of approximately 0.05–0.08 per 100,000 people. They most commonly occur in the fourth to sixth decades of life and are more often diagnosed in men. Given the rarity of these tumors, high-quality evidence to inform the clinical management of spinal MPEs remains scarce. Furthermore, there are few studies of advanced contemporary therapeutics specifically for MPE, although early hypothesis-generating studies exploring the role of molecular diagnostic and therapeutic strategies have begun to emerge [10]. To date, most published data describe single institutional experiences treating spinal MPEs. In general, the consensus optimal treatment strategy is similar to ependymoma at large, wherein maximal safe surgical resection is recommended [11,12,13]. The role of chemotherapy and radiotherapy remains controversial [14,15,16].

To our knowledge, Bates et al. has published the largest study of clinical outcomes for individuals with MPE to date [17]. Utilizing the Surveillance, Epidemiology, and End Results (SEER) database in 2016, the authors described treatment outcomes for all patients with MPE at that time, regardless of primary disease site. Within that cohort, Bates et al. found that receipt of surgery, age of less than 30, and Caucasian race were significantly associated with improved survival. However, receipt of radiotherapy was associated with worse survival, likely owing to treatment selection biases wherein radiotherapy was more often utilized in cases of more aggressive or unfavorable disease.

In this investigation, we aim to build upon prior clinical studies on MPE by utilizing the most recent iteration of the SEER database, which not only reflects the most contemporary treatment strategies but also includes the largest study sample size to date. Furthermore, we aim to focus specifically on outcomes and management strategies for spinal MPEs, as these tumors may reflect a more homogeneous pathophysiology and warrant specific management strategies.

## 2. Materials and Methods

### 2.1. Patient Population

The SEER program of the National Cancer Institute collects information on cancer incidence and survival in the United States starting in 1975. In order to study the outcome of MPE in the modern era, we selected SEER research data from 17 registries spanning 2000 to 2020, which captures approximately 26.5% of the U.S. population [18]. We excluded patients who were not in active follow-up, with disease outside of the spine, and unknown surgical status. Notably, the SEER database does not include radiation dose, fractionation, or radiotherapy technique.

Because the SEER database reports on a national sample of deidentified patients, there is no risk for individuals represented in the study. As such, this study abides by the standards set forth in the Declaration of Helsinki and was considered exempt from review by the MD Anderson Cancer Center Institutional Review Board.

### 2.2. Study Design

In this observational cohort study, several variables from the SEER were included in our analyses. These included the following: age at diagnosis (continuous in years), sex (male or female), race (American Indian/Alaska Native, Asian or Pacific Islander, Black, or White), marital status (married or unmarried), income status (described in categorical fashion as <USD 70,000 or USD 70,000 or more), city population size (described in categorical fashion as <1 million or 1 million or more), and receipt of surgery (yes or no).

Receipt of radiotherapy was not included in this analysis due to limited treatment information and high levels of suspected confounding based upon prior literature utilizing the SEER database specifically for studying ependymomas.

### 2.3. Study Outcomes

The primary outcome of the study, overall survival (OS), was calculated from time of diagnosis to time of death. Additional secondary outcomes of interest included the characterization of patterns of care among the study population and the assessment of changes in treatment paradigms over time.

### 2.4. Statistical Analysis

Normality of distributions of continuous variables was assessed with visual inspection via density plots and quantile–quantile plots and was assessed quantitatively with the Shapiro–Wilk test. All tested variables were not normally distributed (Shapiro–Wilk *p* < 0.001), and thus, non-parametric approaches were used for the remainder of the analysis. Analysis of variance (ANOVA) and chi-square tests were used to characterize differences in variables of interest between cohorts of patients receiving different treatment modalities, for continuous and categorical variables, respectively. Median follow-up time was calculated via the reverse Kaplan–Meier method described by Schemper and Smith, in which being alive at last follow-up was designated as the event of interest while death from any cause was censored [19]. Cox proportional hazards regression analysis was performed to examine the association between all-cause mortality and age at diagnosis, sex, race, marital status, income status, population density, and surgical status. In this analysis, individuals who remained alive at the conclusion of the follow-up period were treated as censored observations. Hazard ratios and *p*-values were calculated and illustrated as a forest plot. All statistical analyses were carried out using R version 4.3.2 (R Foundation for Statistical Computing, Vienna, Austria) with 2-sided testing and a statistical significance threshold of 0.05.

## 3. Results

### 3.1. Study Population and Treatment Characteristics

We identified 1197 patients with spinal MPEs that met inclusion criteria. The population characteristics are summarized in Table 1. Overall, most patients were White (89.9%) with a moderate male predominance (57.5%). The median age at diagnosis [interquartile range (IQR)] was 42 (28–55) years, and the incidence of new diagnoses per year was relatively stable during the data collection period. The majority of patients received definitive surgical resection (95%).

### 3.2. Survival Outcomes

**Figure 1:**
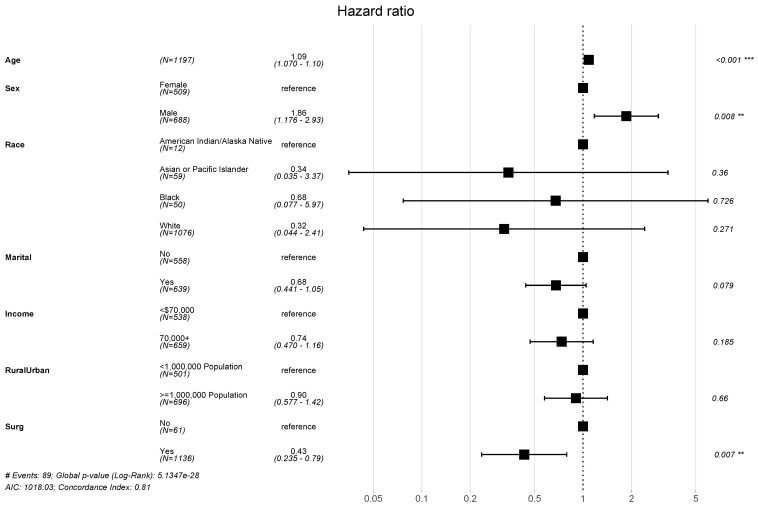
Forest plot describing factors associated with overall survival. Point estimates of hazard ratios (HR) for death are provided with associated 95% confidence intervals; ** and *** denote statistically significant results, assuming P-value thresholds of 0.01, and 0.001, respectively.

A total of 89 all-cause mortality events were identified in this study population, yielding high OS among this population. The estimated 5-, 10- and 15-year OS were 95.8%, 91.4%, and 85.5%, respectively. Results from the multivariable Cox regression are summarized in a forest plot (Figure 1). When adjusting for covariates, age (hazard ratio (HR) = 1.09, [95% confidence interval] = 1.07–1.1, *p* < 0.001), male sex (HR = 1.86 [1.18–2.93], *p* = 0.008), and receipt of surgery (HR = 0.43 [0.24–0.79], *p* = 0.007) emerged as significant predictors of OS.

**Figure 2:**
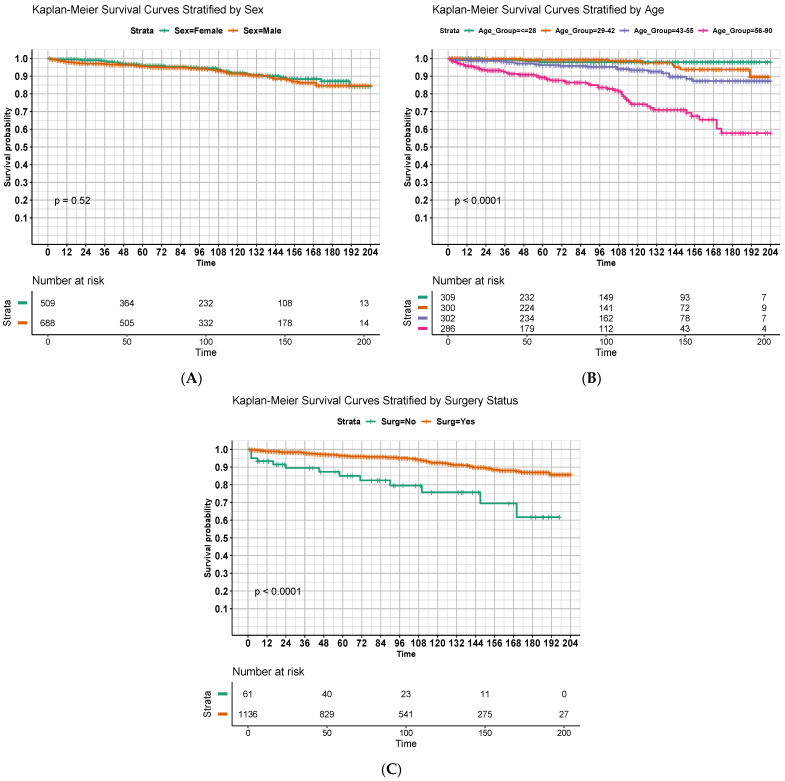
Overall survival of patients in months, stratified by (**A**) sex; (**B**) age quartiles; and (**C**) receipt of surgery. Strata for (**B**) were defined by nearest quartile to allow for near-equal population sizes between groups.

Figure 2 displays OS estimates with respect to months since diagnosis using the Kaplan–Meier method with stratification by significant predictors identified in the multivariable model. Univariate comparisons of curves using the log-rank method are displayed as well. Younger age (Figure 2B, *p* < 0.001) and receipt of surgery (Figure 2C, *p* < 0.001) were again significantly predictive of improved OS, although there was no apparent difference in survival curves based on sex (Figure 2A, *p* = 0.52).

In order to further investigate potential relationships between covariates of interest, we analyzed and compared subpopulation characteristics. Table 2 summarizes population features with stratification by sex and receipt of surgery. Overall, men tended to be diagnosed at younger ages (median 40 vs. 45 years for males and females, respectively, *p* = 0.004). There were no differences in surgical management according to sex. When stratifying by receipt of surgery, individuals who did not undergo surgery tended to be older (median 41 vs. 53 years, respectively, *p* < 0.001). There were no other significant differences in population characteristics when stratified by variables of interest.

## 4. Discussion

In this large population-based study, we observed excellent survival outcomes for individuals with spinal MPE, with an estimated 10-year OS of 91.4% for all patients. We additionally identified several clinical and treatment factors that were predictive of outcomes, including age and receipt of surgery, both of which have been previously described [20]. Interestingly, in this cohort, male sex was also identified as a potential predictor of worse survival in a multivariable model and was associated with younger age at diagnosis compared to females. To the best of our knowledge, we are the first to describe this association between sex and age at presentation, which may imply a unique sex-specific pathophysiology of MPE that could influence prognosis. This finding of demographic differences likely can be extrapolated to the general population, considering the SEER database used in this study encompasses over a quarter of the U.S. population. Furthermore, this study represents the largest cohort to date and thus may contribute unique insights to guide clinical treatment strategies.

Given its rarity, spinal MPE remains a difficult disease to study with robust methodology such as randomized controlled trials. Indeed, the majority of previous investigations describing spinal MPE are single institution retrospective reviews or case series describing management strategies and outcomes for patients with MPEs, often representing only tens of patients or less [21,22,23]. As such, evidence to guide optimal treatment strategies has been mixed and prone to institutional bias. Primary surgical management is universally described as the backbone of therapy and has been shown to consistently and profoundly improve outcomes [13,24,25,26,27,28]. Our data support this notion, as receipt of surgery was associated with improved OS even when accounting for other covariates in a multivariable model.

Evidence for the role of other therapeutic modalities such as radiotherapy is more controversial. Some smaller observational studies, including a joint collaboration between MD Anderson Cancer Center and the Rare Cancer Network, suggest that radiotherapy is associated with improved local disease control, which may be especially beneficial for patients with other high-risk features for local recurrence such as subtotal resection [29,30,31]. Similarly, small series suggest that radiotherapy may be a beneficial adjuvant or salvage strategy for controlling spinal MPEs with anaplastic features [32]. Another retrospective review of 58 patients undergoing surgical resection found a trend toward improved disease control with the utilization of adjuvant radiotherapy for disease with capsular invasion [15]. In a contemporary literature review of adjuvant management strategies for MPE, Jahanbakhshi et al. identified mixed data for adjuvant radiotherapy while confirming maximal surgical resection as the most important therapy for improving patient outcomes [16].

To our knowledge, the largest prior study of MPE to date was the 2016 report from Bates et al. that similarly utilized the SEER dataset to describe outcomes for individuals with MPE [17]. The authors identified 773 patients with MPE (not explicitly limited to the spine) and concluded that receipt of surgery (HR = 0.14, *p* < 0.001), receipt of radiotherapy (HR = 4.06, *p* < 0.001), age less than 30 years (HR = 0.24, *p* = 0.01), and Caucasian race (HR = 0.37, *p* = 0.049) were statistically significant prognostic factors. Interestingly, radiotherapy was significantly associated with worse survival outcomes, which the authors argue was likely representative of incompletely adjusted selection bias, wherein radiotherapy was offered to patients with more adverse features.

Our study contributes uniquely to this landscape for several reasons. First, we utilized an updated cohort from SEER of almost a decade and thus captured almost 50% more patients in our study population, which may therefore provide additional insights and statistical power when compared to the study by Bates et al. [17]. Second, our primary aim was to focus specifically on outcomes for MPEs located in this spine, as these tumors likely possess characteristic pathophysiological characteristics and behavior. We therefore excluded all patients with non-spine disease. Third, to our knowledge, this is the first study of spinal MPE to suggest differential age distributions and potential survival differences among males and females. While we believe this is the first report to suggest such sex-specific associations specifically for MPE, there are prior reports showing similar findings for ependymoma at large, particularly among the pediatric population, providing additional evidence that these trends reflect true underlying pathophysiology. Although our results are very likely biased by some degree of unmeasured confounding, this finding is hypothesis-generating and warrants further investigation.

Although this investigation draws strength as the largest cohort of spinal MPE to date, it is nonetheless limited by several factors related to the study design. First, the SEER database itself has only limited information on many treatment and disease-related factors. There are several variables that would be of interest for this study, such as radiotherapy dose or status for en-bloc resection, and that were unavailable in the dataset and thus could not be considered within our model. Information regarding environmental or occupational exposures could similarly provide hypothesis-generating information regarding disease pathophysiology and prognosis but could not be assessed within this study design. Indeed, given the incomplete information regarding radiotherapy details, we elected to omit radiation from our multivariable model. This decision was informed in part from Bates et al., who found a counterintuitive relationship where radiotherapy was associated with significantly worse survival (HR = 4.06), which potentially arose from a lack of a homogeneous primary site (e.g., spine) and information on radiation modality, radiation dose, and radiographic/pathological factors that led to the decision of choosing adjuvant radiation therapy. Furthermore, there are no detailed data on salvage therapies for disease progression or cause of death, thus representing another potential source of unaccounted confounding, particularly when describing OS outcomes. Secondly, as with any retrospective study design, it is impossible to ascertain causal relationships between treatment variables and outcomes of interest. Nonetheless, we attempted to address confounding through the creation of a multivariable model. Last, although the SEER database reports on a relatively proportion of the US population, it is possible that there are systematic selection biases wherein the individuals captured within the database are not representative of the population at large, thus compromising the generalizability of findings.

## 5. Conclusions

In this SEER database study, we observed excellent overall survival among patients with spinal MPE, with younger age at diagnosis and receipt of surgical resection emerging as significant predictors of improved outcomes. Additionally, our findings suggest a potential association between male sex and worse survival, highlighting a need for further investigation into sex-specific pathophysiological factors influencing prognosis. Despite the limitations inherent in retrospective studies and the limited data available in the SEER database, our study, representing the largest cohort of spinal MPEs to date, contributes important evidence to guide clinical management strategies for this rare tumor type. These results can therefore be translated to improve patient management, education, and counseling. Further research is warranted to better understand the optimal treatment approaches and factors influencing outcomes in patients with spinal MPEs.

## Figures and Tables

**Table 1:** Characteristics of the overall study population. Abbreviations: IQR = interquartile range.

	Count (%)
	*n* = 1197
Age in years (median [IQR])	42.00 [28.00, 55.00]
Sex	
Female	509 (42.5)
Male	688 (57.5)
Year of Diagnosis	81 (6.8)
2004	77 (6.4)
2005	78 (6.5)
2006	67 (5.6)
2007	71 (5.9)
2008	61 (5.1)
2009	76 (6.3)
2010	79 (6.6)
2011	73 (6.1)
2012	63 (5.3)
2013	69 (5.8)
2014	73 (6.1)
2015	69 (5.8)
2016	61 (5.1)
2017	66 (5.5)
2018	71 (5.9)
2019	62 (5.2)
2020	58 (5.1)
Race	
American Indian Alaska Native	12 (1.0)
Asian or Pacific Islander	59 (4.9)
Black	50 (4.2)
White	1076 (89.9)
Marital Status	
Unmarried	558 (46.6)
Married	639 (53.4)
Income	
<USD 70,000	538 (41.9)
USD 70,000 or more	659 (55.1)
City Population Size	
<1 million	538 (44.9)
1 million or more	696 (58.1)
Receipt of Surgery	
No	61 (5.1)
Yes	1136 (94.9)

**Table 2:** Characteristics of the study population, with results stratified by significant predictors of overall survival, including sex and receipt of surgery. Abbreviations: IQR = interquartile range.

	Sex			Receipt of Surgery		
	Female (%)	Male (%)	*p* Value	No Surgery (%)	Surgery (%)	*p* Value
	*n* = 509	*n* = 688		*n* = 61	*n* = 1136	
Age in years (median [IQR])	45.00 [29.00, 57.00]	40.00 [28.00, 53.00]	0.004	53.00 [37.00, 64.00]	41.00 [28.00, 54.00]	<0.001
Sex						
Female	--	--		25 (41)	484 (42.6)	
Male	--	--		36 (59)	652 (57.4)	
Year of Diagnosis			0.624			0.222
2004	35 (6.9)	46 (6.7)		2 (3.3)	79 (7.0)	
2005	28 (5.5)	49 (7.1)		6 (9.8)	71 (6.2)	
2006	36 (7.1)	42 (6.1)		6 (9.8)	72 (6.3)	
2007	24 (4.7)	43 (6.2)		2 (3.3)	65 (5.7)	
2008	32 (6.3)	39 (5.7)		2 (3.3)	69 (6.1)	
2009	26 (5.1)	35 (5.1)		7 (11.5)	54 (4.8)	
2010	32 (6.3)	44 (6.4)		0 (0.0)	76 (6.7)	
2011	30 (5.9)	49 (7.1)		3 (4.9)	76 (6.7)	
2012	35 (6.9)	38 (5.5)		4 (6.6)	69 (6.1)	
2013	29 (5.7)	34 (4.9)		6 (9.8)	57 (5.0)	
2014	33 (6.5)	36 (5.2)		3 (4.9)	66 (5.8)	
2015	31 (6.1)	42 (6.1)		3 (4.9)	70 (6.2)	
2016	25 (4.9)	44 (6.4)		3 (4.9)	66 (5.8)	
2017	29 (5.7)	32 (4.7)		4 (6.6)	57 (5.0)	
2018	36 (7.1)	30 (4.4)		1 (1.6)	65 (5.7)	
2019	25 (4.9)	46 (6.7)		5 (8.2)	66 (5.8)	
2020	23 (4.5)	39 (5.7)		4 (6.6)	58 (5.1)	
Race			0.476			0.631
American Indian Alaska Native	7 (1.4)	5 (0.7)		0 (0.0)	12 (1.1)	
Asian or Pacific Islander	29 (5.7)	30 (4.4)		3 (4.9)	56 (4.9)	
Black	22 (4.3)	28 (4.1)		1 (1.6)	49 (4.3)	
White	451 (88.6)	625 (90.8)		57 (93.4)	1019 (89.7)	
Marital Status			0.466			0.193
Unmarried	244 (47.9)	314 (45.6)		23 (37.7)	535 (47.1)	
Married	265 (52.1)	374 (54.4)		38 (62.3)	601 (52.9)	
Income			0.9			0.613
<USD 70,000	229 (45)	314 (45.6)		25 (41)	513 (45.2)	
USD 70,000 or more	280 (55)	374 (54.4)		36 (59)	623 (54.8)	
City Population Size			0.085			0.18
<1 million	198 (38.9)	303 (44)		20 (32.8)	481 (42.3)	
1 million or more	311 (61.1)	385 (56)		41 (67.2)	655 (57.7)	
Receipt of Surgery			0.907			
No	25 (4.9)	36 (5.2)		--	--	
Yes	484 (95.1)	652 (94.8)		--	--	

## Data Availability

Data are available through request to the corresponding author.
